# Thick Filament Mechano-Sensing in Skeletal and Cardiac Muscles: A Common Mechanism Able to Adapt the Energetic Cost of the Contraction to the Task

**DOI:** 10.3389/fphys.2018.00736

**Published:** 2018-06-14

**Authors:** Gabriella Piazzesi, Marco Caremani, Marco Linari, Massimo Reconditi, Vincenzo Lombardi

**Affiliations:** PhysioLab, University of Florence, Florence, Italy

**Keywords:** cardiac muscle regulation, skeletal muscle regulation, thick filament mechano-sensing, small angle X-ray diffraction, Frank-Starling law, myosin motor, duty ratio

## Abstract

A dual regulation of contraction operates in both skeletal and cardiac muscles. The first mechanism, based on Ca^2+^-dependent structural changes of the regulatory proteins in the thin filament, makes the actin sites available for binding of the myosin motors. The second recruits the myosin heads from the OFF state, in which they are unable to split ATP and bind to actin, in relation to the force during contraction. Comparison of the relevant X-ray diffraction signals marking the state of the thick filament demonstrates that the force feedback that controls the regulatory state of the thick filament works in the same way in skeletal as in cardiac muscles: even if in an isometric tetanus of skeletal muscle force is under the control of the firing frequency of the motor unit, while in a heartbeat force is controlled by the afterload, the stress-sensor switching the motors ON plays the same role in adapting the energetic cost of the contraction to the force. A new aspect of the Frank-Starling law of the heart emerges: independent of the diastolic filling of the ventricle, the number of myosin motors switched ON during systole, and thus the energetic cost of contraction, are tuned to the arterial pressure. Deterioration of the thick-filament regulation mechanism may explain the hyper-contractility related to hypertrophic cardiomyopathy, an inherited heart disease that in 40% of cases is due to mutations in cardiac myosin.

## Introduction

In striated (skeletal and cardiac) muscles, the contractile machinery is organized in sarcomeres, 2-μm long structural units in which two antiparallel arrays of myosin motors from the thick filament generate steady force and shortening by cyclic ATP-driven interactions with the nearby thin actin-containing filaments originating from the opposite extremities of the sarcomere. According to the classical model of regulation of striated muscle, contraction is initiated by the increase of intracellular Ca^2+^-concentration ([Ca^2+^]_i_), induced by membrane depolarization by the action potential, followed by Ca^2+^-dependent structural changes in the regulatory proteins on the thin filament that release the actin sites for binding of the myosin motors (Ebashi et al., 1969; Huxley, 1973; Gordon et al., 2000). However, growing evidence that myosin motors in the resting muscle lie along the surface of the thick filament, folded towards the center of the sarcomere, unable to bind actin (Woodhead et al., 2005; Zoghbi et al., 2008) and hydrolyze ATP (Stewart et al., 2010), raised the question of how the motors can sense the state of the thin filament during activation. Using X-ray diffraction on intact myo-cells from skeletal and cardiac muscles at ID02 beamline of the European Synchrotron (ESRF, Grenoble, France) (Narayanan et al., 2017), a second regulatory mechanism, based on thick filament mechano-sensing, has been identified, which controls the recruitment of myosin motors from the state at rest in relation to the load (Linari et al., 2015; Reconditi et al., 2017).

## Dual Filament Regulation in the Skeletal Muscle

**FIGURE 1:**
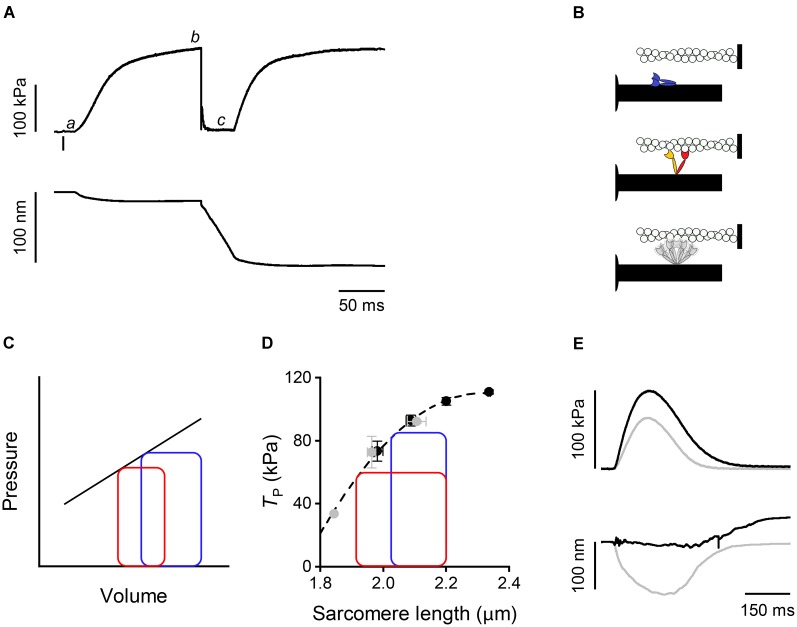
Mechanical responses in skeletal and cardiac muscle and schematic of myosin motor configurations. **(A)** Force (upper panel) and half-sarcomere length change (lower panel) as measured by the striation follower (Huxley et al., 1981) during a tetanic stimulation of a single fiber dissected from frog muscle (at 4°C and SL 2.15 μm). The small bar below the force trace indicates the time of the first stimulus. a: latency period; b: plateau of force attained in isometric conditions; c: phase in which a shortening at *V*_0_ is imposed on the fiber. **(B)** Schematic of the motor conformation in various states: blue, OFF state; red, attached force-generating motor; orange, detached partner of the attached force-generating motor; gray, detached dimer in the ON state. **(C)** Pressure–volume loops with two different diastolic filling (preload): blue, high preload; red, low preload. The straight line describes the end-systolic pressure–end-systolic volume (ESPV) relation. **(D)** Relation between force at the peak of the twitch and sarcomere length in a cardiac trabecula at 2.5 mM Ca^2+^ and 27°C. Gray circles, FE conditions; black circles, LC conditions. The dashed line is the exponential fit to the experimental points. The two loops are ideal pressure–volume loops drawn assuming that two isometric contractions start at the same SL (2.2 μm) and become isotonic at two different levels of force corresponding to two different points in the ESPV relation depending on the blood pressure. **(E)** Force (upper panel) and half-sarcomere length changes (lower panel) during a trabecula twitch under fixed-end (gray) and length clamp (black) conditions at 2.5 mM Ca^2+^ and 27°C.

In a tetanic contraction of skeletal muscle (**Figure 1A**), the thin filament is kept activated by the maintained high level of [Ca^2+^]_i_ induced by repetitive firing of action potentials (Caputo et al., 1994). [Ca^2+^]_i_ raises from the resting level (<10^-7^ M) to a maximum (∼10^-5^ M) within 10 ms from the first action potential, which correspond to the latent period for the mechanical response (**Figure 1A,a**), inducing a rapid structural change in the regulatory troponin-tropomyosin complex on the thin filament that exposes actin sites for binding with myosin motors (Kress et al., 1986; Gordon et al., 2000). Attachment of myosin motors to the actin filament can be structurally characterized using X-ray diffraction in intact muscle cells. By exploiting X-ray interference between the two arrays of myosin motors in each thick filament (Linari et al., 2000), it was found that changes in the fine structure of the M3 meridional reflection, originating from the 14.5-nm axial repeat of myosin motors along the thick filament, indicate a 10-nm movement of the center of mass of the myosin motors during the transition from the resting OFF state, in which they lie on the surface of the thick filament (**Figure 1B**, blue), to the actin-attached state characteristic of the isometric contraction (**Figure 1B**, red) (Huxley et al., 2006; Reconditi et al., 2011, 2014). The structural changes marking thick filament activation, such as the intensity drop of the first myosin layer line reflection (ML1) that records the loss of the three-stranded helical symmetry when myosin motors switch ON (**Figure 1B**, gray), and the ∼1.5% spacing increase of the sixth order meridional reflection (M6) that records the increase in the extension of filament backbone (half-time ∼25 ms), are two times slower than Ca^2+^-dependent thin filament activation, but lead myosin motor attachment and force generation (half-time ∼50 ms; Brunello et al., 2006; Reconditi et al., 2011). However, skeletal muscle can shorten at the maximum velocity (*V*_0_, the velocity under zero load) at the end of the latent period (Lombardi and Menchetti, 1984), when the thin filament is fully activated by Ca^2+^ but the thick filament is still OFF (Linari et al., 2015). This somewhat surprising finding is supported by recent mechanical experiments showing that very few myosin motors (≤3) per half-thick filament are enough to sustain *V*_0_ shortening (Fusi et al., 2017). Most importantly, *V*_0_ shortening imposed at the end of the latent period to prevent force development maintains the OFF structure of the thick filament, even if [Ca^2+^]_i_ is high (Linari et al., 2015). Moreover, if *V*_0_ shortening is superimposed to the plateau of an isometric tetanus (*T*_0_, **Figure 1A,b**), when the thick filament is fully ON, to drop and keep force to zero (**Figure 1A,c**), the OFF state is progressively recovered. Accordingly, the rate of force redevelopment following the end of *V*_0_ shortening is lower the longer the duration of *V*_0_ shortening. Thus, thick filament regulatory state determines the rate of force development and in turn depends on the force acting on the filament by means of a positive feedback that rapidly adapts the number of available motors to the load.

## Dual Filament Regulation in the Cardiac Muscle

Shortly after the discovery of mechano-sensing-based thick filament regulation in skeletal muscle, a similar mechanism was found to operate in the heart (Reconditi et al., 2017). The heart mechanical activity (systole) consists in short periodic contractions (twitches) triggered by single action potentials. During systole the blood is pumped by ventricles into the arterial circulation. In the resting period between two systoles (diastole), the heart is filled by the blood from the venous return. In contrast to skeletal muscle, in cardiac muscle [Ca^2+^]_i_ may not reach the level for full thin filament activation during systole. Consequently, the mechanical response depends on both [Ca^2+^]_i_ and Ca^2+^-sensitivity of the filament (Allen and Kentish, 1985; ter Keurs, 2012), parameters that are under the control of several regulatory systems. These are either intrinsic, like the relation between sarcomere length (SL) and systolic force (a property known as Length Dependent Activation, LDA) (Sagawa et al., 1988; de Tombe et al., 2010), or extrinsic, like neuro-humoral control of the degree of phosphorylation of contractile, regulatory, and cytoskeletal proteins (Hidalgo and Granzier, 2013; Kumar et al., 2015; Kampourakis et al., 2016). LDA is the cellular basis of the Frank–Starling law of the heart that, in its classical formulation, relates the pressure exerted on the blood during the contraction of the ventricle (end-systolic pressure) to its filling during the relaxation (end-diastolic volume) (**Figure 1C**), in this way ensuring the dynamic equilibrium between the two circulatory systems (pulmonary and systemic) driven by two pumps in series. As shown in **Figure 1C**, the loop starting from a larger end-diastolic volume of the ventricle (blue with respect to red) attains a higher afterload during the isovolumetric phase of systole, so that the isotonic shortening terminates with a higher end-systolic pressure intersecting the end systolic pressure-volume (ESPV) relation (black line) at a higher end-systolic volume. In turn, the ESPV relation defines the points at which the contracting cardiac cells are neither lengthening nor shortening, representing the organ correlate of the active tension–length relation at sarcomere level (**Figure 1D**, dashed line) (Arts et al., 1991).

How thick filament mechano-sensing is integrated with the peculiar properties of the heart to modulate its performance was investigated by using X-ray diffraction on electrically paced intact trabeculae, pillar-like multicellular preparations dissected from the internal wall of the ventricle of rat heart. Trabecula attachments to the transducer levers entail an end compliance that during force development in a twitch in fixed-end conditions (FE) causes 10–15% shortening of the sarcomeres (**Figure 1E**, gray trace). Shortening can be prevented by feeding it forward to the loudspeaker motor in the next twitch (length-clamp condition, LC, **Figure 1E**, black trace). The relation between peak-twitch force (*T*_p_) and SL is uniquely determined by the SL at *T*_p_ (black-LC and gray-FE circles lie on the same relation in **Figure 1D**) (Caremani et al., 2016). At the ID02 beamline the possibility to rapidly change the length of the camera from 0.6 to 31 m allows recording of both the meridional reflections from the nanometer-scale assembly of the contractile proteins in the filaments and the micrometer-scale SL. The X-ray signals marking the state of the thick filament show that in diastole the myosin motors are in the same OFF state as that of resting skeletal muscle, while during systole only a fraction of the motors leaves the OFF state, depending on the loading conditions (Reconditi et al., 2017).

## Comparative Analysis of the Mechano-Sensing-Dependent State of the Thick Filament

**FIGURE 2:**
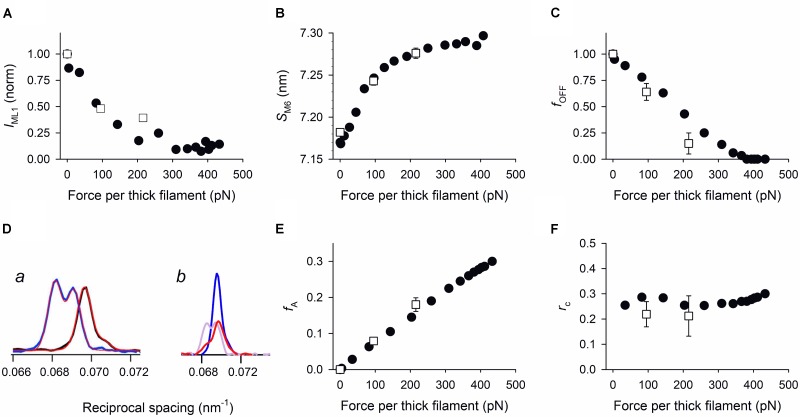
Comparison of force-dependence of the relevant parameters marking the regulatory state of the thick filament. Filled circles: data from force development of isometric tetanus of single muscle fibers; open squares: data from *T*_p_ of trabeculae in FE or LC conditions. Force per thick filament in skeletal muscle fiber and heart trabecula is calculated from the force per cross-sectional area reported in the original papers, taking into account the fractional occupancy of the myofibrils, 0.83 in frog skeletal muscle (Mobley and Eisenberg, 1975) and 0.61 in rat trabeculae (Schaper et al., 1985; Barth et al., 1992), and the filament lattice spacing that is the same in both muscles (Irving et al., 2000; Reconditi et al., 2014). The calculated density of thick filaments is 5.87.10^14^ m^-2^ and 4.31.10^14^ m^-2^ in the skeletal muscle fiber and in the trabecula respectively. **(A)** *I*_ML1,_ intensity of ML1 reflection. Filled circles from Figure 4B in Reconditi et al., 2011; open squares from Figure S2C in Reconditi et al., 2017. **(B)** *S*_M6_, spacing of M6 reflection. Filled circles from Figure 3d in Linari et al., 2015; open squares from Figure 2F in Reconditi et al., 2017. **(C)** *f*_OFF_, fraction of motors in the OFF state. Filled circles from Figure 3D in Reconditi et al., 2011; open squares from Table 1 in Reconditi et al., 2017. **(D)** Comparison of the intensity profiles of the M3 reflection in skeletal muscle (a, from Figure 3C in Reconditi et al., 2011; thick black trace, rest; thick blue trace, *T*_0_; red lines, outputs of the structural model) and cardiac muscle (b, from Figure 2C in Reconditi et al., 2017; blue trace, diastole; violet and red traces, high force and low force twitch, respectively). The intensity profile of the low force twitch (red trace in b) is solely explained by including the contribution from a substantial fraction of the myosin motors in the OFF conformation. **(E)** Fraction of actin-attached motors (*f*_A_). Filled circles and open squares as in **(C)**. **(F)** *r*_c_, duty ratio. Filled circles calculated from filled circles in **(C)** and **(E)**. Open squares from Table 1 in Reconditi et al., 2017.

Does the positive feedback between force and thick filament activation work in the same way in skeletal and cardiac muscles? This question is analyzed in **Figure 2** by comparing the force-dependence of the relevant parameters marking the state of the filament, that is the intensity of ML1, *I*_ML1_ (A), the spacing of M6, *S*_M6_ (B) and the fraction of OFF motors, *f*_OFF_ (C), calculated from the structural model simulation of the intensity and fine structure of the M3 reflection (D) (Reconditi et al., 2011, 2017). The model assumes that the axial mass distribution responsible for the M3 reflection is given by the contribution of three populations: (i) motors in the OFF state lying on the surface of the thick filament folded back towards the center of the sarcomere (blue in **Figure 1B**); (ii) attached force-generating motors (red); (iii) detached motors made by the partners of attached motors (orange), and detached dimers not significantly contributing to the M3 intensity for their large conformational dispersion (gray). In skeletal muscle during isometric tetanus development the changes of both *I*_ML1_ (**Figure 2A**, filled circles) and *S*_M6_ (**Figure 2B**, filled circles) are almost complete when the force on the thick filament has attained ∼220 pN (∼0.5 *T*_0_). *f*_OFF_ (**Figure 2C**, filled circles), instead, drops in proportion to force rise, attaining zero at ∼400 pN. In the trabecula both *I*_ML1_ (**Figure 2A**, open squares) and *S*_M6_ (**Figure 2B**, open squares) change with *T*_p_ in the same way as during force development in the skeletal muscle. Also, *f*_OFF_ (**Figure 2C**, open squares) decreases in proportion to force increase, but with a slightly steeper slope, as if the force feedback for switching motors ON had a larger gain than in skeletal muscle.

Sarcomere-level mechanics allows the comparison to be extended to two functional parameters of the regulatory state of the thick filament: the fraction of actin-attached motors *f*_A_ (**Figure 2E**), and the duty ratio *r*_c_ (the ratio between *f*_A_ and the fraction of motors available for actin interaction, 1-*f*_OFF_) (**Figure 2F**). *f*_A_ increases in proportion to the isometric force in both skeletal (filled circles) and cardiac (open squares) muscles (Brunello et al., 2006; Fusi et al., 2010; Pinzauti et al., 2018). The duty ratio *r*_c_, instead, results independent of the force, and thus of the regulatory state of the thick filament, in both muscle fibers (filled circles) and trabeculae (open squares). This result is not surprising: in fact, in either case, the increase in *f*_A_ occurs without change in the strain per attached motor and thus, in terms of Huxley, 1957 two-state model, without change in the fraction of the ATPase cycle time each motor spends attached to the actin. We conclude that force controls the regulatory state of the thick filament modulating the number of available motors and not the kinetics of motor attachment to actin (Irving, 2017).

## Molecular Bases of Thick Filament Mechano-Sensing

The structural and functional parameters related to thick filament regulation reported in **Figure 2** depend on the force in a similar way in skeletal and cardiac muscle, through the action of a common, still unknown, mechano-sensor. The finding that the switching ON of the motors is accompanied by an increase in the backbone axial periodicity one order of magnitude larger than that due to filament compliance, strongly supports the idea that the common mechanism is based on the stress-sensitivity of the intermolecular–intramolecular interactions determining the helical packing of myosin motors in the OFF state. These interactions are generated not only within the myosin molecules (head–head and head–tail interactions responsible for what is called the interacting head motif, IHM; Alamo et al., 2008), but also with other thick filament proteins like titin and myosin binding protein C (MyBP-C) that are assembled so as to match the 43-nm helical periodicity of the myosin molecules (Rome et al., 1973; Labeit et al., 1992). The stress-dependent disruption of these interactions would determine the release of myosin motors from the surface of the filament with loss of the helical order and 1.5% extension of the backbone (Huxley and Brown, 1967; Irving, 2017). A role as a mechano-sensor has been attributed to titin, which spans the whole half-sarcomere, connecting the Z-line at the end of the sarcomere with the tip of the myosin filament and then running, bound to the surface of the thick filament, up to the M-line at the center of the sarcomere. Titin can transmit the stress to thick filament also in the resting sarcomere, when no motors are attached to actin, as recently demonstrated in relaxed skinned fibers from mammalian skeletal muscle (Fusi et al., 2016), in which, however, the features of the resting state of the intact cell are only partially preserved (Xu et al., 2009; Caremani et al., 2017). The hypothesis of titin as a mechano-sensor is further supported by the finding that titin stiffness increases with the increase in [Ca^2+^] (Labeit et al., 2003), but is weakened by the evidence that the titin mediated stress of the thick filament has been proved only at SL > 2.8 μm. MyBP-C is bound with its C-terminal to the backbone of the thick filament in the central one-third of the half-sarcomere (C-zone) and extends from the thick filament to establish, with its N-terminal, dynamic interactions, controlled by the level of phosphorylation, with either the actin filament or the rod-like S2 domain of the myosin (Moos et al., 1978; Rybakova et al., 2011; Pfuhl and Gautel, 2012). In cardiac MyBP-C, a supplementary N-terminal domain, the C0 domain, dynamically interacts with either the actin or the regulatory light chain (RLC) in the myosin head. MyBP-C is the most likely interfilament signaling protein able to affect the IHM (Kampourakis et al., 2014; Harris et al., 2016; Kensler et al., 2017). In the intact muscle fiber the resting viscosity (likely related to inter-filamentary links) disappears at the end of latent period, when the fiber becomes able to shorten at *V*_0_ (Lombardi and Menchetti, 1984). Noteworthy in the cardiac cell the development of *V*_0_-shortening is much slower and is completed when force attains 50% of the maximum twitch force (de Tombe and ter Keurs, 1992), suggesting different dynamics for the disappearance of the internal load.

Another actor in thick filament regulation, specific of the cardiac myocyte, is the basic elevated degree of phosphorylation of the RLC (40%, Toepfer et al., 2013); accordingly, disruption of the OFF state depends on RLC phosphorylation (Kampourakis and Irving, 2015). The phosphorylation-dependent tuning by MyBP-C and RLC of the thick filament state in cardiac muscle might explain the larger gain in the force feedback that switches motors ON in cardiac muscle with respect to skeletal muscle (**Figure 2C**).

Mutations in the aminoacids on the surface of the S1 domain are causative of hypertrophic cardiomyopathy (HCM), an inherited heart disease characterized by thickening of the ventricular wall that diminishes the relaxation capacity and ventricular filling. In the folded IHM state, the surface of the S1 domain presents binding sites for the proximal S2 domain of the myosin and for the MyBP-C (Spudich, 2015; Alamo et al., 2017; Trivedi et al., 2018) and mutations in this region (the myosin mesa) would weaken the interactions responsible for the IHM, leading to the hyper-contractility associated with HCM. The demonstration of stress-sensing in thick filament of cardiac muscle opens a new scenario in which the HCM-causing mutations in the myosin mesa produce hyper-contractility by reducing the force threshold of the switch that controls the fraction of motors in the ON state.

## Role of Thick Filament Mechano-Sensing in Skeletal and Cardiac Muscles

In spite of the strict similarities of thick filament mechano-sensing in skeletal and cardiac muscles, the mechanism is integrated in peculiar ways with the function of these muscles. In the skeletal muscle, during the high firing frequency that sustains maximum tetanic force *T*_0_, mechano-sensing in the thick filament activation speeds up force development during high load contraction (Linari et al., 2015). However, voluntary movements during the physiological activity of skeletal muscle may imply lower firing frequencies and consequently sub-tetanic forces that can be even lower than 0.5 *T*_0_ (Macefield et al., 1996). In this case, thick filament mechano-sensing provides partial activation (**Figures 2A–C**), revealing a supplementary energetic gain in the tuning of contraction by the firing frequency of the nerve. In addition, thick filament mechano-sensing explains the reduction of ATP utilization below the value expected from solution kinetics measurements if the contraction occurs at very low load (Homsher et al., 1981; Fusi et al., 2017).

In a heartbeat, the whole contraction is submaximal and the force generated during systole varies in a range within which, as shown by open squares in **Figure 2**, a given fraction of motors remains in the OFF state. In this case, the positive feedback between force and thick filament activation operates to adapt the switched ON motors to the load, independent of the diastolic sarcomere length. The LC and FE twitches in **Figure 1E** approximate the conditions of the left ventricle beating against a high (LC twitch) and a low (FE twitch) aortic pressure. In turn, the ESPV relation of the left ventricle (**Figure 1C**, continuous line) is the organ correlate of the active force–SL relation (**Figure 1D** dashed line). The two ideal loops in **Figure 1D** represent contractions that start at 2.2 μm SL and become isotonic when the force attains the level identified by the intercept on the relation (blue: high load, red: low load). At organ level they correspond to two pressure-volume loops with the same preload (end-diastolic volume) and different afterloads (aortic pressures), providing a new view of the Frank–Starling mechanism: independent of the diastolic filling of the ventricle, the recruitment of myosin motors and thus the energetic cost of systole is tuned to the load, that is to the aortic pressure.

## Perspectives

Future work, aimed at identifying the molecular basis of thick filament mechano-sensing, acquires particular relevance in cardiac muscle in relation to the Ca^2+^-dependent thin filament activation and to the destabilizing action of the phosphorylation of the proteins contributing to the OFF state of the motor. The discovery of mechano-sensing in the thick filament implies that the hyper-contractility accompanying HCM-causing mutations in these proteins would result not only from a dysregulation of their degree of phosphorylation but also from an alteration of the gain of the positive feedback between force on the thick filament and motor recruitment. Understanding the molecular basis of the mechano-sensing controlling the regulatory state of the thick filament is a prerequisite in drug development for specific therapeutic interventions.

## Author Contributions

GP, MC, ML, MR, and VL wrote and edited the manuscript.

## Conflict of Interest Statement

The authors declare that the research was conducted in the absence of any commercial or financial relationships that could be construed as a potential conflict of interest.
